# Nanoengineered-based delivery systems to modulate CD4^+^ T cell responses in cancer: emerging paradigms in cancer immunotherapy

**DOI:** 10.3389/fphar.2025.1643791

**Published:** 2025-08-11

**Authors:** Nekhat Shahreen, Anukrati Agnihotri, Asfi Rizwan, Faizul Hasan, Mohd Danish Ansari, Zarif Mohamed Sofian, Nur Akmarina B. M. Said, Kenneth K. W. To, Syed Mahmood

**Affiliations:** ^1^ School of Pharmaceutical Education and Research (SPER), Jamia Hamdard University, New Delhi, India; ^2^ Metro College of Health Sciences and Research, Greater Noida, Uttar Pradesh, India; ^3^ Department of Pharmaceutical Technology, Faculty of Pharmacy, Universiti Malaya, Kuala Lumpur, Malaysia; ^4^ Department of Pharmaceutical Life Sciences, Faculty of Pharmacy, Universiti Malaya, Kuala Lumpur, Malaysia; ^5^ School of Pharmacy, Faculty of Medicine, The Chinese University of Hong Kong, Hong Kong SAR, China; ^6^ Faculty of Medicine, Universiti Malaya Research Centre for Biopharmaceuticals and Advanced Therapeutics (UBAT), Universiti Malaya, Kuala Lumpur, Malaysia; ^7^ Centre of Advanced Materials (CAM), Faculty of Engineering, Universiti Malaya, Kuala Lumpur, Malaysia; ^8^ Faculty of Pharmaceutical Sciences, Chulalongkorn University, Bangkok, Thailand

**Keywords:** CD4^+^ T-cells, cancer immunology, nanoformulation, novel drug delivery systems, tumor microenvironment

## Abstract

**Background:**

CD4^+^ T-cells play a pivotal role in cancer immunology, functioning as both tumor-suppressing and tumor-promoting agents depending on their differentiation and cytokine profiles. Targeting CD4^+^ T-cells with novel drug delivery systems, particularly nanoparticle-based formulations, offers a promising approach to enhancing antitumor immune responses while minimizing systemic toxicity.

**Objective:**

This review aims to explore the immunological significance of CD4^+^ T-cells in cancer and their modulation using novel drug delivery systems. The focus is on understanding CD4^+^ T-cell subtypes, their functional roles in tumor progression and suppression, and the application of novel drug delivery systems to selectively regulate these cells.

**Methods:**

A comprehensive analysis of CD4^+^ T-cell subsets, including Th1, Th2, Th17, Tregs, and Tfh, was conducted, along with their immunological roles in cancer. Various nanoparticle platforms, including liposomes, polymeric nanoparticles, dendrimers, gold, silver, and mesoporous silica, were evaluated for their ability to target CD4^+^ T-cells.

**Results:**

Novel drug delivery systems demonstrate significant potential in selectively modulating CD4^+^ T-cell responses. Liposomes and polymeric nanoparticles efficiently transport cytokines, antigens, as well as immunological modulators to CD4^+^ T-cells, enhancing antitumor immunity. Notably, MHC II-coated nanoparticles expanded antigen-specific CD4^+^ T-cells, while mRNA nano vaccines activated CD4^+^ and CD8^+^ responses.

**Conclusion:**

Novel drug delivery systems provide a versatile platform for precise CD4^+^ T-cell modulation in cancer therapy, enhancing antitumor responses while reducing toxicity. Future advancements should focus on overcoming biological barriers, improving targeting, and optimizing clinical translation.

## 1 Introduction

Cancer immunology has become a foundation of modern oncology, highlighting the complex relationships between the host immune system and cancerous cells (Finn, 2012). The concept of immune surveillance, by which immune cells identify and eradicate transformed cells, is crucial for tumour prevention (Kim, 2007). However, cancer cells frequently develop defenses against immune system recognition and elimination, leading to a phenomenon known as immunoediting, which promotes tumour progression (Kim, 2007; Ribatti, 2017). In recent years, immune checkpoint inhibitors and other immunotherapeutic approaches, adoptive T-cell transfer, and cancer vaccines have revolutionized treatment strategies by reactivating and enhancing anti-tumour immune responses (Parsonidis and Papasotiriou, 2022; Perica et al., 2015). Despite their success, these therapies are not universally effective, and a large number of patients either show no response at all or eventually become resistant, underscoring the need for more targeted treatments and a deeper understanding of immune regulation (Kazemi et al., 2016; Klener et al., 2015). Among the key players in tumour immunology, Traditionally, CD4^+^ T-cells have been acknowledged for their role as helper cells that support cytotoxic CD8^+^ T-cell responses and B-cell activation (Alberts et al., 2002; Bevan, 2004). However, emerging evidence reveals that CD4^+^ T-cells are far more versatile. Beyond their helper function, certain CD4^+^ T-cell subsets possess direct cytotoxic activity and perform important functions in controlling other immune cells and modifying the tumor microenvironment (TME) by secreting cytokines. For example, Th1 and Th17 subsets are associated with potent anti-tumour effects, while regulatory T-cells (Tregs) often contribute to immune suppression and tumour tolerance (Accogli et al., 2021; Li et al., 2020). The dynamic balance between these subsets can significantly influence the ability of the immune system to control tumour growth and metastasis. Alongside these developments, nanotechnology has emerged as a valuable tool to enhance the efficacy and precision of cancer immunotherapies. Nanoformulations offer multiple advantages, including improved solubility and stability of therapeutic agents, targeted delivery, and controlled release kinetics (Jeevanandam et al., 2016) (Han and Park, 2023). These properties enable more effective modulation of the immune system with reduced systemic toxicity. Specifically, Tumor antigens, adjuvants, immune checkpoint inhibitors, or cytokines can all be delivered using nanoparticles. Directly to antigen-presenting cells or T-lymphocyte subsets, including CD4^+^ T-cells (Goldberg, 2019; Gao et al., 2019). Innovations such as ligand-functionalized nanoparticles, pH-responsive carriers, and biomimetic nanocarriers further enhance the ability to selectively modulate immune responses within the tumour microenvironment, thereby potentiating CD4^+^ T-cell activation and function (Niculescu and Grumezescu, 2022; Gowd et al., 2022).

In parallel with advancements in immunology, the evolution of Novel Drug Delivery Systems (NDDS) has significantly improved the accuracy and effectiveness of cancer treatments. By facilitating the regulated and site-specific distribution of therapeutic drugs, NDDS plays a crucial role in targeted therapy by reducing systemic toxicity and optimising drug accumulation at the tumour site (Theivendren et al., 2024). Liposomes, dendrimers, polymeric nanoparticles, and lipid-based nanoparticles have all demonstrated considerable potential among the many NDDS platforms (Hassanzadeh-Khanmiri et al., 2025). Their use in immunotherapy has been transformed by surface modification techniques, such as PEGylation for longer circulation time, ligand conjugation (e.g., antibodies, peptides, or aptamers) for receptor-mediated targeting, and pH- or enzyme-sensitive coatings for tumour microenvironment responsiveness (Maurya and Tyagi, 2024). These modified nanocarriers can be made to target certain immune cells, such as CD4^+^ T-cells, or elements of the cancer microenvironment, increasing the therapeutic index of checkpoint inhibitors, cytokines, and immunomodulators (Saeed et al., 2019). Among these methods, ligand-functionalized polymeric nanoparticles have become especially well-known because of their biocompatibility and adaptable surface chemistry, which enable improved tumour penetration and precise engagement with immunological targets (Anwar et al., 2024).

This review offers a unique perspective by integrating the immunological complexity of CD4^+^ T-cell subsets with the latest advancements in NDDS. While prior reviews have extensively discussed CD4^+^ T-cell biology, PEGylation, and checkpoint inhibitors separately, this article distinguishes itself by focusing on how nanocarriers such as liposomes, polymeric nanoparticles, dendrimers, and inorganic nanoparticles can be specifically designed to modulate subsets of CD4^+^ T-cells (e.g., Th1, Th17, Tregs) within the TME. The review also emphasizes novel approaches like MHC II-coated nanoparticles for *ex vivo* CD4^+^ T-cell expansion, dual CD4^+^/CD8^+^ activation through mRNA nanovaccines, and functionalized nanocarriers for the targeted delivery of cytokines or checkpoint inhibitors (Lee et al., 2021). By combining cutting-edge nanotechnology with immunological insights, this work highlights the transformative potential of nano-immunotherapy for targeting CD4^+^ T-cells in cancer treatment. It provides a comprehensive overview of CD4^+^ T-cells’ diverse roles in tumor suppression and promotion, as well as innovative NDDS-based strategies to enhance therapeutic outcomes. Moreover, the review explores challenges and opportunities for the clinical application of these novel strategies, filling a critical gap in existing literature that typically treats these fields separately (Shen et al., 2024).

## 2 CD4^+^ T-Cells in cancer immunology: a comprehensive exploration

### 2.1 Overview of CD4^+^ T-cells in the immune system and cancer microenvironment

Helper T-cells are a major class of T-lymphocytes and play an important role in orchestrating adaptive immune responses. They determine the antigenic peptides linked with the major histocompatibility complex class II (MHC-II) molecules on dendritic cells, macrophages and B-cells, which are referred to as antigen-presenting cells (APCs),2 whose main role is to present these peptides to the major histocompatibility complex class II (MHC-II) molecules. These cells are then activated and differentiated into a variety of functional subtypes, each with specialized immune modulating activities (Borst et al., 2018; Speiser et al., 2023). CD4^+^ T-cells play a dual role in cancer immunology, either supporting immune evasion or enhancing anti-tumor immunity, depending on the surrounding cytokine milieu and cellular interactions within the TME. Their distinct functional differences, from driving cytotoxic responses to establishing an immunosuppressive niche, highlight their significance as both therapeutic targets and prognostic biomarkers in oncology.

### 2.2 Subtypes of CD4^+^ T-cells and their functional implications in cancer immunity

**FIGURE 1:**
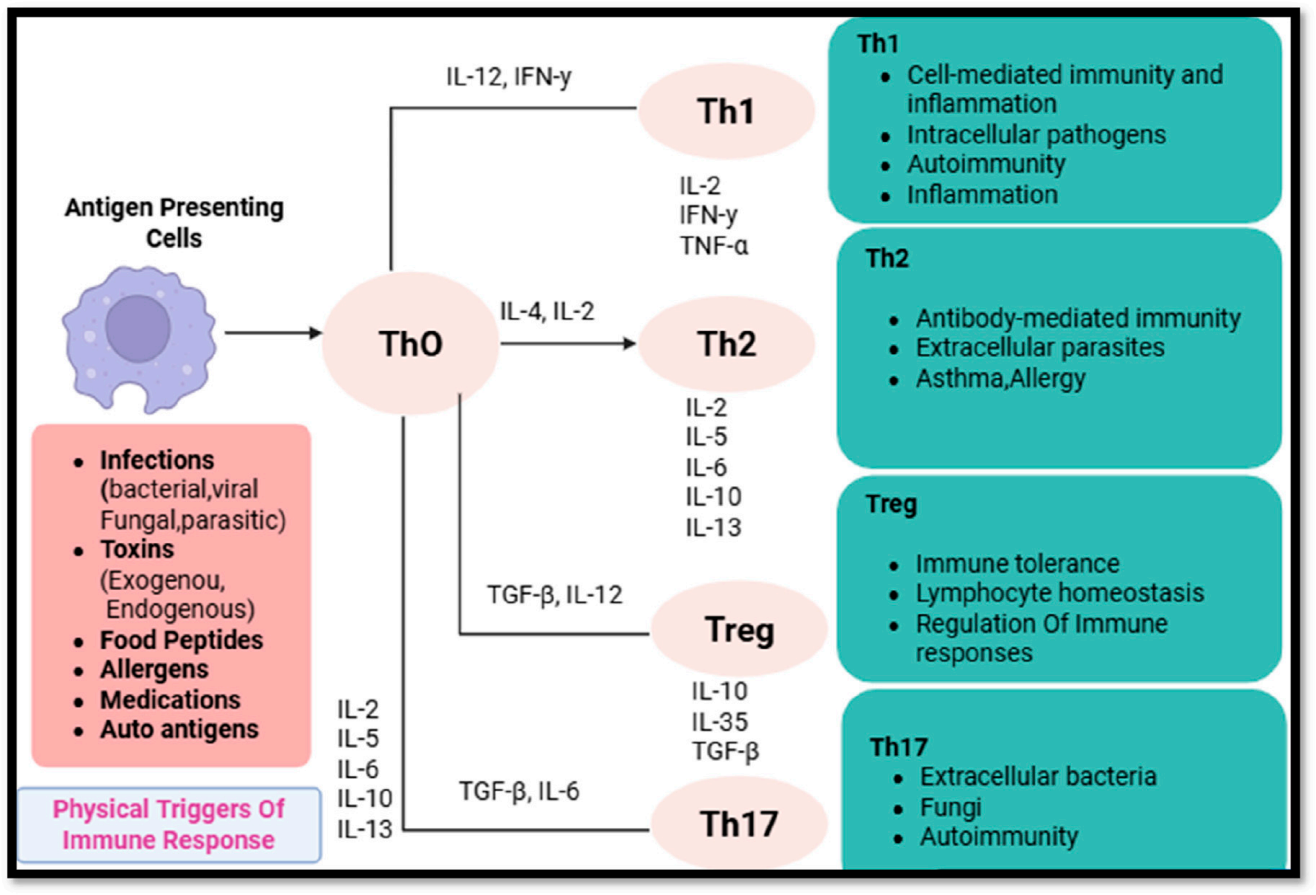
Differentiation and functions of CD4^+^ T-cell subsets in response to antigenic stimuli.

The differentiation of CD4^+^ T-cells into specialized subsets is governed by specific transcription factors, cytokine secretion patterns, and epigenetic modifications (Speiser et al., 2023). These subsets include helper T-cells (Th1, Th2, Th17, and Tfh), regulatory T-cells (Tregs), and effector/memory T-cells, each of which modulates tumor immunity in unique ways (Chatzileontiadou et al., 2021). CD4^+^ T-cell subset distinction and functions in reaction to antigens, depicted in Figure 1.

#### 2.2.1 Helper T-cells (Th1, Th2, Th17, Tfh) and their contrasting roles

Th1 Cells: Th1 cells, A hallmark of these cells is the T-bet transcription factor, which mediates the secretion of interferon-gamma (IFN-γ) and tumor necrosis factor-alpha (TNF-α), that, in turn, augments the cytolytic function of CD8^+^ T-cells and localizes natural killer (NK) cells. NK cells can recognize and kill tumor cells independent of antigen, and they secrete IFN-γ.26-28. In addition, IFN-γ promotes expression of MHC-I on tumor cells, thereby enhancing antigen presentation and cross-presentation of proinflammatory recognition (Haabeth et al., 2011). A strong Th1 response in the TME is correlated with good clinical outcomes because of the persisting inflammatory microenvironment that prevents tumors from spreading.

Th2 Cells: In contrast, Th2 cells, under the influence of the transcription factor GATA3, secrete IL-4, IL-5, IL-10, and IL-13, which skew immune responses towards humoral immunity and eosinophil activation (Jacenik et al., 2023). Th2-mediated cytokine profiles have been implicated in immune evasion by fostering an anti-inflammatory microenvironment that suppresses CD8^+^ T-cell cytotoxicity and promotes macrophage polarization towards the tumor-supportive M2 phenotype. Consequently, Th2-dominated immune responses are frequently correlated with poor prognostic outcomes across various malignancies.

Th17 Cells: Governed by the transcription factor RORγt, Th17 cells produce IL-17, IL-21, and IL-22, cytokines that exhibit a dual role in cancer biology. Acute IL-17 signaling can enhance anti-tumor immunity by recruiting neutrophils and promoting inflammation, yet chronic IL-17 exposure fosters immune tolerance, angiogenesis, and stromal remodeling, ultimately contributing to tumor progression and metastasis.

Tfh Cells: Follicular helper T-cells (Tfh), defined by Bcl-6 expression, primarily reside within germinal centers, where they assist in B-cell maturation and antibody production. Although their role in cancer immunity is less well-characterized, emerging evidence suggests that tumor-infiltrating Tfh cells may influence anti-tumor humoral responses, potentially affecting immunotherapeutic efficacy.

#### 2.2.2 Regulatory T-cells (Tregs) and their immunosuppressive influence in tumors

Tregs, identified by FoxP3 expression, constitute a specialized subset of CD4^+^ T-cells dedicated to maintaining immune homeostasis by suppressing excessive inflammatory responses (Togashi et al., 2019). However, within the TME, Tregs contribute to immune evasion through several mechanisms:

• Secretion of immunosuppressive cytokines such as IL-10 and transforming growth factor-beta (TGF-β), which dampen effector T-cell activation and promote tissue remodeling.
• The expression of immunological checkpoint molecules that suppress T-cell receptor (TCR) signaling and prevent effector T-cell proliferation, such as cytotoxic T-lymphocyte-associated protein 4 (CTLA-4) and programmed cell death protein 1 (PD-1).
• Metabolic competition for IL-2, depriving effector T-cells of a critical survival factor, thereby limiting their expansion and functionality.

Increased infiltration of Tregs in solid tumors correlates with poor prognostic outcomes, as their suppressive activity facilitates immune escape and tumor progression. Targeting Tregs through selective depletion or functional modulation remains a promising strategy in cancer immunotherapy.

#### 2.2.3 Effector vs. memory CD4^+^ T-cells: implications for long-term cancer immunity

Effector CD4^+^ T-cells are short-lived and primarily function by secreting cytokines that drive immediate immune responses (Barnaba, 2022). In contrast, memory CD4^+^ T-cells, which can be categorized into central memory (Tcm) and effector memory (Tem) subsets, play a pivotal role in long-term tumor surveillance:

• Tcm Cells: Residing in secondary lymphoid organs, Tcm cells exhibit rapid proliferative capacity upon antigen re-exposure and sustain long-lasting immune responses.
• Tem Cells: Circulating in peripheral tissues, Tem cells mediate immediate effector responses upon antigen recognition, thus playing a critical role in controlling tumor recurrence.

The presence of tumor-specific memory CD4^+^ T-cells has been linked to improved survival outcomes, highlighting their significance as potential targets for cancer vaccines and adoptive cell therapies.

### 2.3 CD4^+^ T-cells and the TME

#### 2.3.1 Cellular interactions and immune modulation

CD4^+^ T-cells dynamically interact with various immune cells within the TME, shaping the overall immune landscape of tumors (Accogli et al., 2021). These interactions include:

CD8^+^ T-Cells: CD4^+^ T-cells enhance CD8^+^ T-cell priming and expansion through IL-2 and IFN-γ secretion. However, in immunosuppressive TMEs, exhausted CD4^+^ T-cells fail to provide adequate support, leading to diminished CD8^+^ cytotoxic responses.

Macrophages: Th1-driven signaling promotes M1 macrophage polarization, enhancing anti-tumor activity, whereas Th2 and Tregs contribute to M2 macrophage differentiation, facilitating immune evasion.

Dendritic Cells: CD4^+^ T-cells modulate dendritic cell function by influencing antigen presentation and cytokine secretion. In tumor settings, tolerogenic dendritic cells may limit effective T-cell activation, contributing to immune escape.

#### 2.3.2 Cytokine and chemokine regulation of CD4^+^ T-cells in tumors

Cytokines such as IL-2 (promoting T-cell proliferation), IFN-γ (enhancing antigen presentation), and IL-10 (mediating immune suppression) critically regulate CD4^+^ T-cell function in cancer. Furthermore, chemokines such as CCL5 and CXCL9 dictate CD4^+^ T-cell infiltration patterns within tumors, ultimately influencing the strength and nature of the anti-tumor immune response (Zhang Y. et al., 2020).

#### 2.3.3 Tumor immune evasion mechanisms targeting CD4^+^ T-cells

Tumors employ a variety of mechanisms to evade CD4^+^ T-cell-mediated immune responses. One key strategy involves the upregulation of PD-L1 and other immune checkpoint ligands, which suppress T-cell activation and function. Additionally, tumors secrete immunosuppressive cytokines such as TGF-β and IL-10, which influence CD4^+^ T-cell differentiation, favoring regulatory T cells (Tregs) and Th2 phenotypes that contribute to an immunosuppressive TME. Furthermore, tumors recruit immunosuppressive cell populations, including Tregs and myeloid-derived suppressor cells (MDSCs), which further dampen effective anti-tumor immunity. The various subtypes of CD4^+^ T cells and their immunological roles in cancer are summarized in Table 1 (Dobrzanski, 2013).

**TABLE 1:** Subtypes of CD4^+^ T-cells and their Immunological Roles in Cancer.

CD4^+^ T-cell subtype	Key cytokines produced	Immunological role in cancer	References
Th1	IFN-γ, TNF-α, IL-2	Promotes anti-tumor immunity by activating cytotoxic T lymphocytes and macrophages	Zhu and Paul (2008)
Th2	IL-4, IL-5, IL-13	Supports tumor progression by inhibiting Th1 responses and promoting pro-tumorigenic inflammation	DeNardo and Coussens (2007)
Th17	IL-17, IL-22, IL-23	Can have dual roles: promotes inflammation aiding anti-tumor immunity but can also contribute to tumor progression	Kryczek et al. (2007)
Treg (Regulatory T-cells)	IL-10, TGF-β	Suppresses anti-tumor immune responses, facilitating tumor immune evasion	Curiel et al. (2004)
Tfh (Follicular helper T-cells)	IL-21, CXCL13	Supports B-cell responses in germinal centers; role in cancer immunity is context-dependent	Crotty (2014)
Th9	IL-9	Exhibits anti-tumor immunity through mast cell activation and modulation of the TME	Lu et al. (2012)
Th22	IL-22, TNF-α	Contributes to tissue repair but may promote tumorigenesis in some cancers	Korns et al. (2011)

## 3 Mechanisms of CD4^+^ T-cells in tumor progression and suppression

**FIGURE 2:**
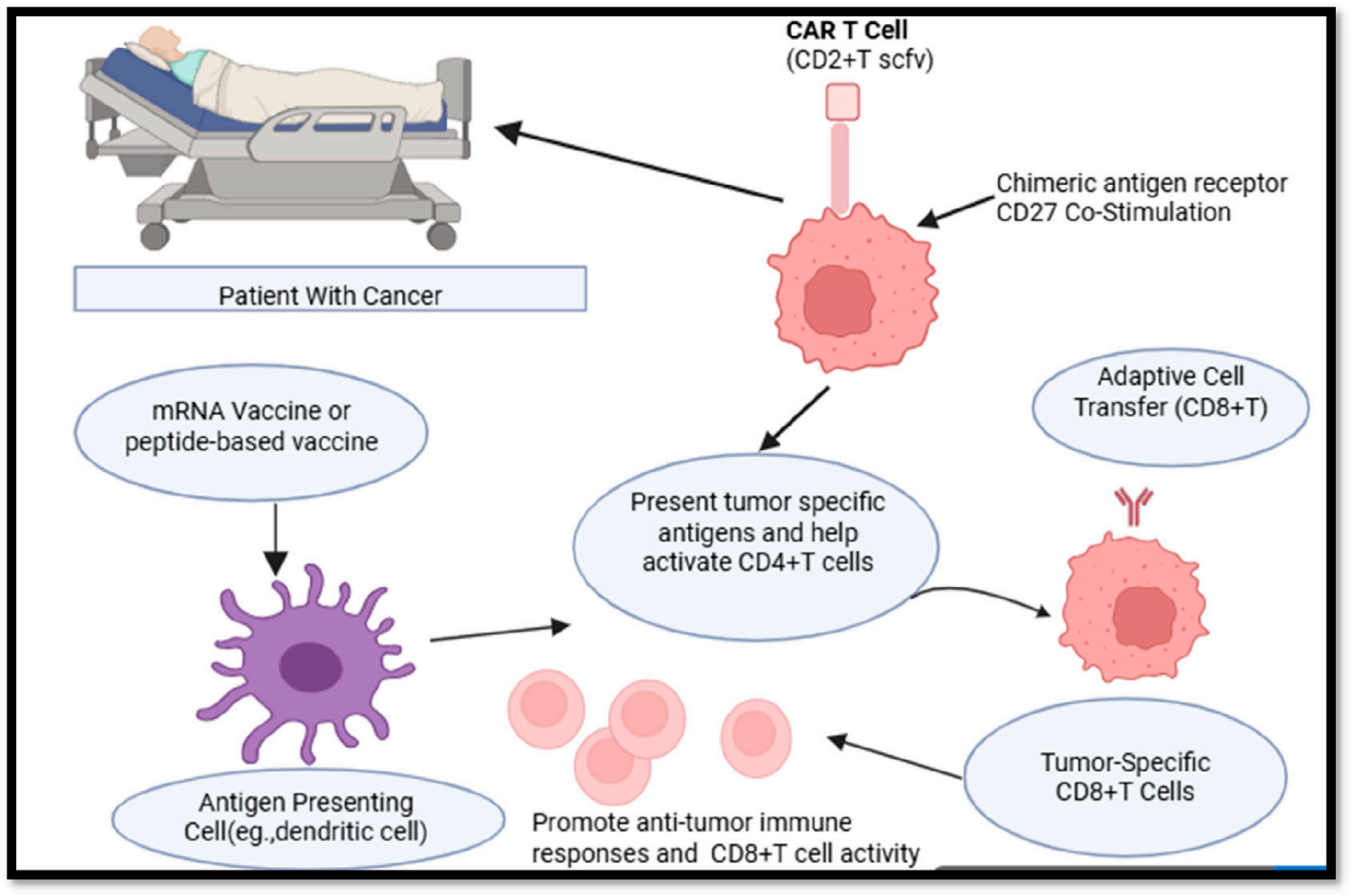
Role of CD4^+^ T Cells in enhancing cancer immunotherapy strategies.

CD4^+^ T-cells play a dual role in cancer immunology, acting as both tumor-promoting and tumor-suppressing agents depending on their differentiation, cytokine profiles, and interactions with other immune components (Ostroumov et al., 2018). The majority of cancer immunotherapy techniques may benefit from including CD4^+^ activation techniques. T cell assistance or its byproducts. Peptides, or the DNA or mRNA that codes for them, are used in Both MHC class I and MHC class II epitopes should be included in therapeutic vaccinations to guarantee that CD4^+^ Activation of T cells in the answer. To overcome tumor-associated immune suppression, agonistic CD27 antibodies can be used to modulate the signaling pathways of CD4^+^ T-cells, effectively enhancing their helper functions and promoting robust T cell-mediated anti-tumor responses. These antibodies may also function in concert with PD1 inhibition. DC subsets that can prime CD4^+^ T cells and provide aid signals to cytotoxic T lymphocytes (CTLs) are essential for dendritic cell (DC)-based immunization. As an alternative, XC-chemokine receptor 1 (XCR1)+ may be the target of antigens and activation signals. DCs that migrate and stay in lymph nodes employing antibody-conjugates that are unique to this lineage of DC139. CD4^+^ should be included into adoptive cell treatment. T lymphocytes to aid at the location of the tumor or make advantage of preprogrammed assistance CD8^+^ T cells. When assistance signals are sent, transferred CTLs need to be increased. Cells that carry the signaling pattern of suitable co-stimulatory receptors may be used in chimeric antigen receptor (CAR) T cell therapy to replicate the delivery of assistance. The activation of conventional type 1 DCs (cDC1s) and their subsequent interaction with CD4^+^ T cells may be supported by treatments that elicit a type I interferon response, such as radiation or stimulator of interferon genes protein (STING) agonists (Figure 2).

### 3.1 Tumor-promoting CD4^+^ T Cells: Tregs and Th2 roles

Regulatory T-cells (Tregs) and T-helper 2 (Th2) cells are implicated in tumor progression due to their immunosuppressive functions. Tregs, identified by the expression of FOXP3, exert their immunosuppressive effects primarily through the secretion of inhibitory cytokines such as interleukin-10 (IL-10) and transforming growth factor-beta (TGF-β) (Whiteside, 2012). These cytokines suppress effector T-cell proliferation, impair antigen-presenting cell (APC) function, and promote the development of an immune-privileged environment conducive to tumor growth. Furthermore, Tregs contribute to metabolic competition by consuming high levels of interleukin-2 (IL-2), thereby depriving effector T-cells of a critical survival signal.

Th2 cells promote a tumor-supportive milieu by secreting IL-4, IL-5, and IL-13, which facilitate alternative macrophage activation and suppress cytotoxic immune responses. The predominance of Th2-driven immunity is associated with poor prognosis in several malignancies, as it fosters an immunosuppressive microenvironment that inhibits effective tumor clearance.

#### 3.1.1 Role of tregs in immune suppression

Tregs exert immunosuppressive effects through multiple mechanisms, including cytokine-mediated inhibition, metabolic disruption, and direct cell-cell interactions. The secretion of IL-10 and TGF-β not only suppresses CD8^+^ cytotoxic T lymphocytes (CTLs) but also inhibits the maturation and antigen-presenting capacity of dendritic cells (DCs) (Palomares et al., 2010). Tregs also engage in contact-dependent suppression via CTLA-4-mediated inhibition of co-stimulatory signaling on APCs, further dampening anti-tumor immune responses. Experimental depletion of Tregs in preclinical cancer models leads to enhanced T-cell infiltration and improved tumor control, highlighting their role as key mediators of immune evasion.

#### 3.1.2 CD4^+^ T-cell exhaustion and dysfunction

Prolonged antigen exposure within the TME leads to the progressive dysfunction and exhaustion of CD4^+^ T-cells. Exhausted T-cells are characterized by the sustained upregulation of immune checkpoint receptors, including programmed cell death protein-1 (PD-1), T-cell immunoglobulin and mucin domain-containing protein 3 (TIM-3), and lymphocyte activation gene-3 (LAG-3) (Miggelbrink et al., 2021; Xiao et al., 2022). T-cell exhaustion is characterized by the co-expression of inhibitory receptors on CD4^+^ T cells, including PD-1, CTLA-4, LAG-3, and TIM-3. A progressive loss of effector functions, such as decreased proliferative capacity, decreased production of important cytokines like IL-2, IFN-γ, and TNF-α, and impaired cytotoxic activity, are characteristics of this exhausted phenotype (Raziorrouh et al., 2014). Long-term antigenic stimulation in the TME causes these functional deficits, which are frequently exacerbated by immunosuppressive signals and metabolic stress. Consequently, worn-out CD4^+^ T cells are less able to coordinate efficient antitumor immunity or sustain cytotoxic CD8^+^ T-cell responses (Xiao et al., 2022). Especially in solid tumors where chronic antigen exposure is common, this dysfunction poses a significant challenge to cancer immunotherapy. Importantly, immune checkpoint blockade therapies, like anti-PD-1 and anti-CTLA-4 antibodies, have been developed as a result of the identification of this exhaustion program (Retnakumar et al., 2023). These therapies are intended to revitalize exhausted T cells and restore their functional potential. Although CD8^+^ T cells received most of the early attention from ICB, mounting data shows that CD4^+^ T-cell renewal is essential for mediating long-lasting and potent immunotherapy responses (Wang et al., 2022).

### 3.2 Tumor-suppressing CD4^+^ T-cells

#### 3.2.1 Th1-mediated anti-tumor responses

Th1 cells play a pivotal role in anti-tumor immunity by orchestrating CD8^+^ T-cell responses and enhancing antigen presentation. The hallmark cytokines of Th1 cells, including interferon-gamma (IFN-γ) and tumor necrosis factor-alpha (TNF-α), facilitate macrophage activation, upregulate major histocompatibility complex (MHC) expression on tumor cells, and potentiate CTL-mediated cytotoxicity (Ghiringhelli et al., 2004). The presence of Th1-skewed immunity is often associated with improved clinical outcomes in various cancers, as it promotes sustained anti-tumor immune responses and tumor cell apoptosis.

#### 3.2.2 Th17 cells and immune activation

Th17 cells, characterized by the production of interleukin-17 (IL-17), contribute to immune activation by recruiting neutrophils and modulating inflammatory responses. However, their role in cancer is context-dependent (Kim et al., 2013). In some tumor settings, Th17 cells enhance immune surveillance and drive anti-tumor inflammation. Conversely, in certain malignancies, chronic Th17-driven inflammation can support tumor progression by fostering an immunosuppressive niche. The dualistic nature of Th17 responses underscores the complexity of CD4^+^ T-cell-mediated immune modulation in cancer.

### 3.3 Signaling pathways in CD4^+^ T-cell-mediated cancer immunity

#### 3.3.1 PI3K/AKT, JAK/STAT, NF-κB pathway

The differentiation, survival, and effector activity of CD4^+^ T-cells are regulated by a variety of signaling pathways (Hwang et al., 2020). The phosphoinositide 3-kinase (PI3K)/AKT signaling axis is essential for the regulation of T-cell metabolism, proliferation, and survival (Mayer and Arteaga, 2016). In the context of cancer, PI3K activation enhances Treg function and promotes immunosuppression, whereas inactivating this pathway can restore effector T-cell responses and enhance anti-tumor immunity.

The Janus kinase/signal transducer and activator of transcription (JAK/STAT) pathway is integral to the differentiation of CD4^+^ T-cells and the signaling of cytokines (Leonard and Lin, 2000). STAT1 activation induces Th1 differentiation and IFN-γ production, whereas STAT3 signaling induces Th17 differentiation and IL-17 production. An disparity between” pro- and anti-tumor CD4^+^ T-cell subsets can be the consequence of dysregulation of this pathway, which can affect the progression of cancer and the effectiveness of therapy.

Inflammation and T-cell activation are both dependent on the nuclear factor kappa B (NF-κB) pathway. It facilitates the transcription of genes that are involved in the regulation of the immune system, cytokine production, and T-cell survival (Lawrence, 2009). Depending on the context, the activation of NF-κB within the TME can either enhance anti-tumor immunity or facilitate immune evasion.

#### 3.3.2 Checkpoint regulation and CD4^+^ T-Cell exhaustion

The inhibitory checkpoint receptors PD-1, cytotoxic T-lymphocyte-associated protein 4 (CTLA-4), and LAG-3 serve as critical regulators of CD4^+^ T-cell exhaustion (Fu et al., 2020). These molecules limit excessive immune activation but also contribute to immune suppression in cancer. Therapeutic blockade of PD-1 and CTLA-4 has demonstrated significant efficacy in reinvigorating exhausted T-cells and enhancing anti-tumor responses. Combining checkpoint inhibitors with strategies that promote CD4^+^ T-cell differentiation into effector subsets holds promise for improving the efficacy of cancer immunotherapies.

## 4 Nanoformulation-based approaches for targeting CD4^+^ T-cells in cancer therapy

### 4.1 Rationale for nanoformulation-based CD4^+^ T-cell therapy

**FIGURE 3:**
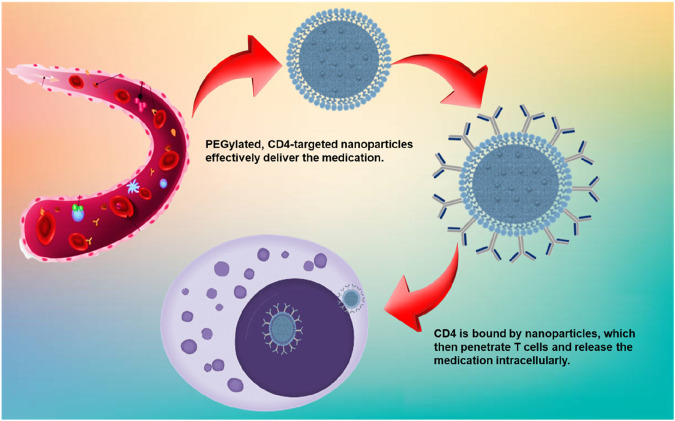
Schematic representation of a nanoformulation-based drug delivery system targeting CD4^+^ T-cells.

Nanoformulations provide an effective and well-regulated means of adjusting CD4^+^ T-cell responses in cancer immunotherapy. Nanocarriers enhance the bioavailability of drugs, shield therapeutic agents from degradation, and facilitate targeted delivery to CD4^+^ T cells, thereby minimizing off-target effects (Peer et al., 2007). Nanocarriers shield immunomodulators from enzymatic breakdown in the blood Figure 3. Functionalized nanoparticles provide selective uptake by CD4^+^ T cells, enhancing therapeutic efficacy. Nanoformulations have the capability to modulate Th1, Th17, and Treg responses for better tumor control (Park et al., 2013).

### 4.2 Types of nanocarriers for CD4^+^ T-cell modulation

#### 4.2.1 Liposomes

**FIGURE 4:**
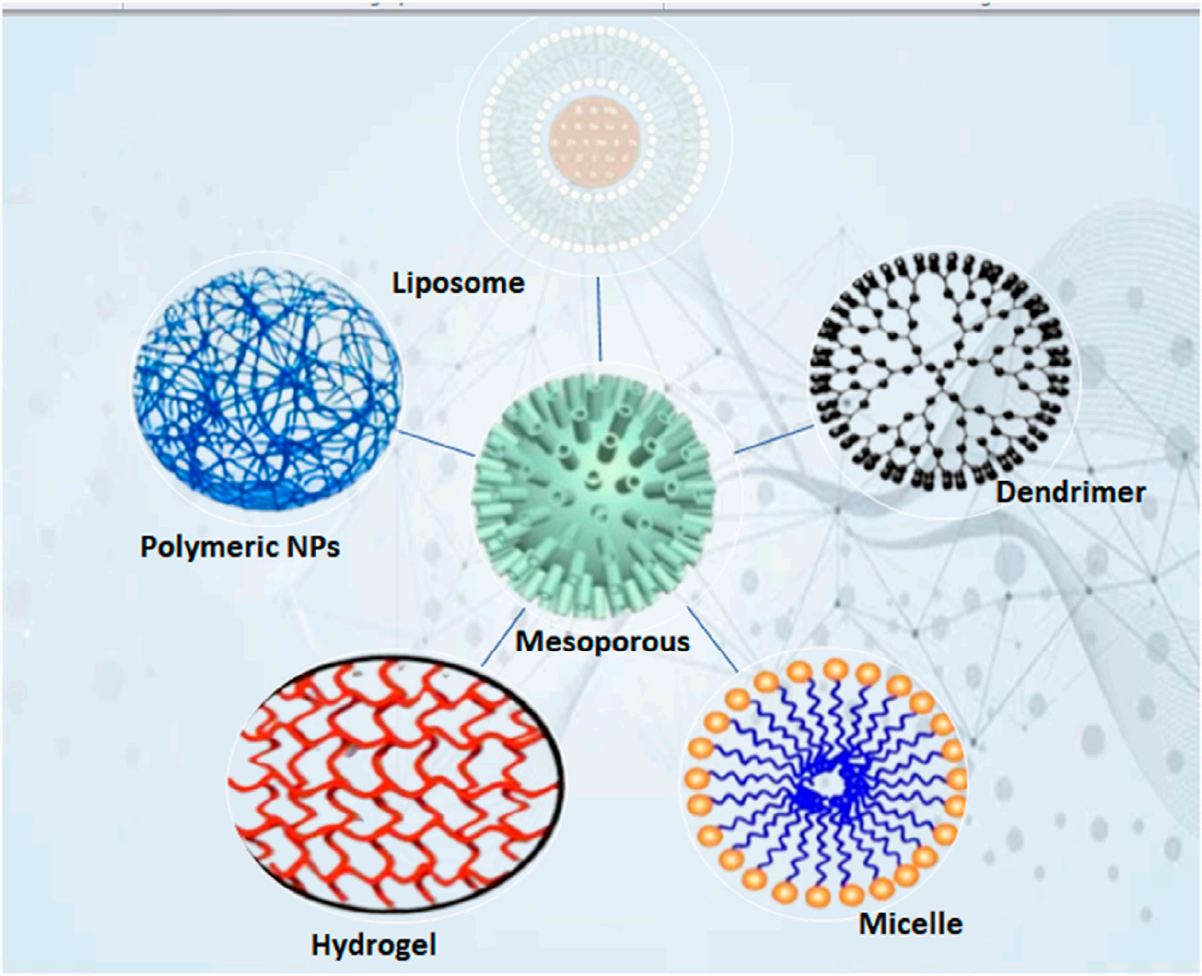
Illustration of the various classes of nano-based novel drug delivery systems.

Phospholipid-based vesicles known as liposomes can encapsulate both hydrophilic and hydrophobic therapeutic agents, safeguarding them from enzymatic degradation and improving their targeted delivery to CD4^+^ T cells. Due to their structural flexibility and capacity to emulate biological membranes, they serve as excellent carriers for immunomodulatory treatments such as cancer immunotherapy, autoimmune disease management, and vaccine creation (Allen and Cullis, 2013). Liposomes safeguard enclosed medications or antigens from being broken down in the blood, thereby improving their therapeutic half-life (Figure 4). The use of functionalized liposomes enhances the uptake by antigen-presenting cells (APCs), which leads to a more efficient activation of CD4^+^ T cells. Liposomes can be designed for drug release that is sensitive to pH, temperature, or enzymes, enabling exact adjustment of CD4^+^ T-cell activation (Lee and Thompson, 2017).

#### 4.2.2 Polymeric nanoparticles

Due to their low toxicity, biodegradability, and FDA approval, poly(lactic-co-glycolic acid) (PLGA) nanoparticles are among the most widely used polymeric carriers for targeted immune modulation. Research indicates that PLGA nanoparticles containing tumor antigens or immune adjuvants can foster antigen-specific CD4^+^ T-cell proliferation and cytokine release, thereby intensifying the Th1-mediated anti-tumor response. With regard to a slow and sustained release of IL-12 or IFN-γ, PLGA nanoparticles can be designed in such a way that they promote long-term activation of CD4^+^ T cells within TME. PLGA nanoparticles that carry both immune checkpoint inhibitors (such as anti-PD-L1) and cytokines enhance tumor regression mediated by CD4^+^ T cells (Danhier et al., 2012).

#### 4.2.3 Dendrimers

Dendrimers, which are highly branched polymers at the nanoscale with precise size, shape, and surface characteristics, serve as outstanding carriers for targeted drug delivery, vaccine formulations, and immunomodulatory therapy Due to their high drug-loading capacity and tunable surface chemistry, Poly(amidoamine) (PAMAM) dendrimers have garnered interest in T-cell-targeted immunotherapies (Mendes et al., 2017). PAMAM dendrimers linked to TLR agonists or IL-12 greatly enhance Th1 polarization, resulting in elevated levels of TNF-α and IFN-γ.By enhancing the activation of CD4^+^ T-cells, tumor clearance is improved with the use of functionalized PAMAM dendrimers that encapsulate immune checkpoint inhibitors (such as anti-PD-1 and anti-CTLA-4) (Huai et al., 2019).

#### 4.2.4 Gold and silver nanoparticles

Gold nanoparticles (AuNPs) and silver nanoparticles (AgNPs) are commonly utilized because of their distinctive immunomodulatory characteristics, compatibility with biological systems, and capacity to improve antigen presentation. The activation of dendritic cells is boosted by the conjugation of tumor antigens with gold nanoparticles, resulting in more robust priming of CD4^+^ T cells. AuNPs that are functionalized with CpG oligonucleotides enhance Th1 cytokine responses, thereby improving anti-tumor immunity (Grzelczak et al., 2008). Silver nanoparticles promote IL-12 secretion in dendritic cells, resulting in improved Th1 differentiation. AgNPs show selective cytotoxic effects on tumor cells, diminishing immunosuppressive factors and improving CD4^+^ T-cell function (Huai et al., 2019).

#### 4.2.5 Inorganic nanoparticles (mesoporous silica, iron oxide)

Due to their large surface area, adjustable pore size, and ability for controlled drug release, mesoporous silica nanoparticles (MSNs) are valuable carriers for immunotherapies. Immune-stimulatory cytokines (IL-2, IL-15) loaded in MSNs facilitate targeted activation of CD4^+^ T-cells within the TME. MSNs can be engineered for the simultaneous delivery of tumor antigens and immune adjuvants, which boosts CD4^+^ T-cell proliferation (Mamaeva et al., 2013). Iron Oxide Nanoparticles (IONPs) for Th1 Immunity and MRI Tracking In the realm of cancer immunotherapy, iron oxide nanoparticles (IONPs) offer both therapeutic and diagnostic benefits. IONPs encourage the polarization of macrophages towards an M1 phenotype, enhancing CD4^+^ T-cell activation. IONPs enable the real-time monitoring of immune cell movement during T-cell therapy (Shestovska et al., 1252).

### 4.3 Functionalization of nanoparticles for targeted CD4^+^ T-Cell therapy

#### 4.3.1 Surface modifications for CD4^+^ targeting

Nanoparticles linked with certain monoclonal antibodies (mAbs) boost their selectivity for CD4^+^ T cells. Anti-CD4-functionalized nanoparticles exhibit selective binding to CD4^+^ T cells, which enhances antigen-specific activation and drug delivery. T-cell receptor (TCR) activation is brought on by anti-CD3-coated nanoparticles, which enhances immunological responses and proliferation (Lo et al., 2013). Bispecific antibody-conjugated nanoparticles that target both CD4^+^ and tumor-associated antigens enhance immune synapse formation and amplify T-cell cytotoxicity (Qin et al., 2024). Immuno stimulatory cytokines can be added to nanoparticles to modify their function and development of CD4^+^T cells. IL-2-functionalized nanoparticles promote T-cell expansion and survival, thereby extending their anti-tumor activity. IL-2 attached to polymeric nanoparticles preferentially encourages the growth of effector T-cells over regulatory T-cells, enhancing Th1 responses (Raker et al., 2020).

#### 4.3.2 PEGylation and ligand-based targeting

Opsonization and the mononuclear phagocyte system’s premature clearance are minimized through the use of PEGylated liposomes and polymeric nanoparticles. PEGylation enhances the biodistribution of nanoparticles, leading to increased tumor accumulation and extended immune activation. Excessive PEGylation over a long duration may hinder immune recognition; approaches such as reversible PEG coatings (e.g., cleavable PEG linkers) are under investigation to achieve a balance between stability and immunogenicity (Blanco et al., 2015). Targeting Based on Ligands By attaching to particular receptors found on CD4^+^ T cells, nanoparticles that are functionalized with ligands enhance selectivity. Folic acid-decorated nanoparticles preferentially attach to activated CD4^+^ T cells that express folate receptors, thereby improving targeted delivery (Table 2). Transferrin-coated nanoparticles enhance the uptake of CD4^+^ T-cells via receptor-mediated endocytosis, thus promoting effective drug delivery (Soe et al., 2019).

**TABLE 2:** Types of NDDS used in CD4^+^ T-cell modulation and their advantages.

Nanocarrier type	Composition	Advantages	References
Liposomes	Phospholipid bilayers	– Biodegradable and compatible with biological systems – Improved drug stability and absorption – Targeted delivery through alterations to the surface	Lee and Thompson (2017)
Polymeric Nanoparticles (PNPs)	PLGA,Chitosan or PEGylated polymers	- Controlled and sustained release of medication - Effective antigen presentation - Promotes CD4^+^ T-cell proliferation	Danhier et al. (2012)
Dendrimers	Highly Branched synthetic polymers	- High capacity for drug loading - Accurate regulation of surface functionalization - Enhanced immune response modulation	Mendes et al. (2017)
Gold Nanoparticles (AuNPs)	Metallic gold core	– Boosts the presentation of antigens – Cytokine production is enhanced – Low toxicity and customizable dimensions	Grzelczak et al. (2008)
Silver Nanoparticles(AgNPs)	Metallic silver core	- Regulates immune reactions - Shows anti-cancer and antimicrobial properties - Enhances the activation of CD4^+^ T-cells	Huai et al. (2019)
Mesoporous Silica Nanoparicles (MSNs)	Silica-based porous structure	- Significant capacity for drug loading - Regulated release of medication - Improved delivery to immune cells	Mamaeva et al. (2013)
Iron Oxide Nanoparticles (IONPs)	Superparamagnetic iron oxide core	- Strengthens Th1 immunity - MRI-traceable for live tracking - Enhances T-cell activation	Shestovska et al. (1252)
Erythrocyte Membrane–Coated Nanoparticles (EMCNPs)	ICG (photothermal agent) and gambogic acid	RES clearance decreases and systemic circulation extends as a result of CD47 “self” signals on RBC membranes	Wang et al. (2018)
Hybrid/Multifunctional Nanoparticle Systems	Magnetic FeO_4_ core is loaded with the photothermal dye indocyanine green (ICG)	Homotypic targeting: uses cancer membrane proteins to bind ID8 tumor cells specifically Long circulation and immune evasion through CD47 derived from red blood cells	Xiong et al. (2021)

## 5 Applications of nano formulations in CD4^+^ T-cell-based cancer therapy

### 5.1 Nanoparticle-based drug delivery for CD4^+^ T-cell activation and regulation

#### 5.1.1 Delivery of cytokines and immune modulators

Nanoparticles provide a flexible means for the targeted transport of cytokines and immunomodulators to CD4^+^ T cells, facilitating accurate immune response adjustment with reduced systemic toxicity (Peer et al., 2007). For CD4^+^ T-cells to proliferate and survive, IL-2 is necessary, especially Th1 and Treg subsets. Nanoparticles loaded with IL-2 enhance Th1-biased CD4^+^ responses, boosting IFN-γ and IL-2 secretion while improving cytotoxic activity against tumor cells (Malek, 2008). IL-12 is a strong immunostimulatory cytokine that drives Th1 differentiation and boosts IFN-γ production. By boosting CD4^+^ T-cell activation and antigen presentation, nanoparticles loaded with IL-12 (such as liposomes or hydrogels) trigger a robust anti-tumor immune response (Trinchieri, 2003). IFN-γ operates downstream of IL-12 and IL-2 signaling, playing a crucial role in macrophage activation, MHC upregulation, and anti-tumor effects. IFN-γ-loaded nanoparticles can be administered either intratumorally or systemically to aid in tumor rejection by enhancing CD4^+^ T-cell function and facilitating CTL priming (Schroder et al., 2004). Nanoparticles that allow for the controlled or sustained release of cytokines provide significant immunological benefits. These include avoiding CD4^+^ T-cell exhaustion, sustaining therapeutic cytokine concentrations in the TME, achieving spatially targeted immune modulation, and minimizing the necessity for repeated dosing—all of which contribute to improved safety, efficacy, and patient compliance (Blanco et al., 2015).

#### 5.1.2 Encapsulation of tumor antigens for vaccine development

Encapsulating tumor-associated antigens (TAAs) like TRP-1 or gp100 peptides within nanoparticles (NPs) represents a promising approach for eliciting antigen-specific CD4^+^ T-cell responses. These nanosystems safeguard the antigen against enzymatic degradation, boost its delivery to antigen-presenting cells (APCs), and enhance lymph node trafficking—essential for triggering adaptive immunity (Liu et al., 2014). Immunostimulatory adjuvants like CpG oligodeoxynucleotides (TLR9 agonists) or Poly I:C (TLR3 agonist) can be co-formulated with nanoparticle vaccines. These adjuvants enhance dendritic cell (DC) maturation and improve antigen processing and presentation through MHC-II pathways, which is essential for CD4^+^ T-cell priming and Th1 polarization (Takahashi et al., 2009).

### 5.2 Nano vaccines for enhancing CD4^+^ T-cell responses

#### 5.2.1 Peptide-based nano vaccines

Nanoparticles loaded with peptides safeguard them against enzymatic breakdown while enhancing their lymphatic transport and delivery to antigen-presenting cells (APCs). These nanoparticles improve MHC class II presentation by dendritic cells (DCs), resulting in heightened activation of CD4^+^ T-cells and their differentiation into Th1 effector cells (Buss et al., 2020). Nano-vaccines that are based on peptides are frequently combined with TLR agonists like CpG (TLR9) or MPLA (TLR4). This combination skews responses towards Th1 immunity and boosts IFN-γ production. These vaccines also aid in the formation of immunological memory, thereby ensuring long-term protection against tumor recurrence through the promotion of CD4^+^ T-helper memory subsets (Chehelgerdi et al., 2023).

#### 5.2.2 mRNA and DNA nanovaccines

mRNA nanovaccines, which are encapsulated in lipid nanoparticles (LNPs), carry the genetic instructions for tumor-associated antigens (TAAs) and immunostimulatory proteins. These are translated *in situ* to elicit responses from both CD4^+^ and CD8^+^ T-cells. LNPs shield mRNA from degradation, enhance efficient endosomal escape, and aid in its delivery to dendritic cells and lymphoid tissues (Pardi et al., 2018). DNA vaccines that utilize electroporation for delivery or are encapsulated in polymeric nanoparticles (like PLGA, PEI) encode for TAAs that are expressed endogenously, leading to persistent antigen presentation. These platforms enhance the CD4^+^ T-cell support for cytotoxic T lymphocytes (CTLs), resulting in improved tumor cell destruction (Xu et al., 2020).

### 5.3 Combination strategies: nano formulations with other therapies

#### 5.3.1 Chemo-immunotherapy using nanoparticles

DNA vaccines that utilize electroporation for delivery or are encapsulated in polymeric nanoparticles (like PLGA, PEI) encode for TAAs that are expressed endogenously, leading to persistent antigen presentation. These platforms enhance the CD4^+^ T-cell support for cytotoxic T lymphocytes (CTLs), resulting in improved tumor cell destruction. These systems lead to Damage-associated molecular patterns (DAMPs) and tumor antigens are released as a result of immunogenic cell death (ICD), which promotes dendritic cell maturation and CD4^+^ T-cell priming (Lang et al., 2024). This method converts “cold” tumors (those without immune infiltration) into “hot” tumors characterized by increased T-cell recruitment, elevated MHC expression, and enhanced immune visibility. In addition, using nanoparticles for targeted chemotherapy delivery minimizes systemic toxicity and maintains the viability of immune cells, especially CD4^+^ T-cells, which are generally vulnerable to drug-induced apoptosis (Mizrahy et al., 2017).

#### 5.3.2 Radiation therapy combined with nano-immunotherapy

RT (radiation therapy) brings about death of immunogenic cells, which emits antigens linked to tumors that are able to be captured by APCs. However, due to the immune response produced by RT alone is frequently insufficient in the immunosuppressive TME (Demaria et al., 2015). Immunomodulators like STING agonists, cytokines, or checkpoint inhibitors can be delivered directly to irradiated tumors using nanoparticles, thereby boosting immune activation (Min et al., 2015). A striking instance concerns the use of nanoparticles to deliver anti-CD47 antibodies alongside radiotherapy. This approach encourages macrophages to phagocytose irradiated tumor cells, enhances antigen cross-presentation, and ultimately boosts the infiltration and activity of CD8^+^ and CD4^+^ T-cells (Boone et al., 2022). Crucially, nanoparticles allow for the spatial and temporal coordination of immunotherapy delivery with RT-induced immune priming, a strategy that has proven to enhance therapeutic efficacy and diminish immune-related adverse events (Ngwa et al., 2018).

#### 5.3.3 Checkpoint inhibitors and nanoparticles

Immune checkpoint inhibitors (ICIs), including anti-PD-1, anti-PD-L1, and anti-CTLA-4 antibodies, have revolutionized cancer immunotherapy by activating cytotoxic T lymphocytes against tumors (Topalian et al., 2015). Nevertheless, the systemic use of these agents is commonly associated with immune-related adverse effects (irAEs), including colitis., pneumonitis, and endocrinopathies, resulting from off-target immune activation. Furthermore, their effectiveness is restricted by their inadequate tumor penetration and brief half-life within the TME, particularly in poorly immunogenic or “cold” tumors (Postow et al., 2018). With the progression of research, nanotechnology is becoming recognized as a crucial facilitator of next-generation checkpoint immunotherapy (Table 3). It provides multifunctional platforms capable of enhancing drug delivery, adjusting the tumor immune landscape, and combining with additional therapies like radiotherapy and vaccinations (Irvine and Dane, 2020).

**TABLE 3:** Overview of nanoformulation designs targeting CD4^+^ T-cell immunotherapies.

Study S.No.	Nanoparticle type	Antigen/target	Adjuvant	Key immune outcome	References
1	Mannose functionalised liposomes	HPV E7 peptide	MPLA (TLR4 agonist)	Targeted DC uptake; promoted CD4^+^ T-cell activation; reduced tumor growth	Yu et al. (2021)
2	Peptide-based nanogels	Melanoma -linked antigens	Polyinosinic:Polycytidylic acid	Induced antigen-specific CD4^+^ T cells; enhanced Th1 cytokine production	Haist et al. (2022)
3	STING-activating lipid nanoparticles	Tumor neoantigens	STING agonist (Cgmp)	CD4^+^ T-cell activation worked together with the CD8^+^ response, leading to improved tumor control	Liu et al. (2022)
4	Layer-by-layer polymeric nanoparticles	Myelin oligodendrocyte	None	Expansion of CD4^+^ Treg specific to the antigen; stopping autoimmune inflammation	Yeste et al. (2012)
5	Polymeric nanoparticles (PLGA)	Tumor antigens	TLR7/8 agonist (R848)	Enhanced TME infiltration and enhanced CD4^+^ and CD8^+^ T-cell activation	Lin et al. (2025)
6	Polyethylene-coated gold nanoparticles (AuNPs)	OVA (Ovalbumin)	CpG oligonucleotide	Elevated cytokine release (IL-2, IFN-γ) and CD4^+^ T-cell activation; more robust DC maturation	Zhou et al. (2016)
7	Lipid-based nanoparticles	OVA	MPLA (TLR4 agonist)	Th1-biased response; robust CD4^+^ T-cell proliferation	Chamcha et al. (2019)
8	Anti-CD4^+^ antibody-conjugated nanogels	CD4^+^ T cells via receptor targeting	Payload (e.g., siRNA)	Enabled the transfer of siRNA to CD4^+^ T cells and altered the function of CD4^+^	Cevaal et al. (2021)
9	CD4^+^-targeted polymer/lipid hybrid nanoparticles	CD4^+^ receptor	Payload	Targeting CD4^+^ T cells specifically and enhancing payload delivery	Fang et al. (2022)
10	CD4^+^ T-cell membrane-coated nanoparticles	HIV gp120/infected cells	None	HIV suppression by viral neutralization and autophagy-like mechanisms	Zhang et al. (2020b)
11	CHP (cholesteryl–pullulan) nanocomplex	HER2 tumor protein	CD4^+^ helper epitopes	CD4^+^ and CD8^+^ T-cell responses were induced; early human trials demonstrated safety	Wells et al. (2024)
12	Self-assembling peptide-hydrogel nanoparticles	Mixed tumor epitopes (gp100, MART-1)	None	Strong Th1 CD4^+^ and CD8^+^ responses; useful in models of melanoma in mice	Wells et al. (2024)
13	Amphiphilic lipid–peptide neoantigen NPs (PNVAC)	Patient-specific neoantigens	CD4^+^/8^+^ epitopes	Long-lasting memory CD4^+^/CD8^+^ T-cell responses; improved cancer patient outcomes	Wells et al. (2024)
14	Liposomes co-encapsulating helper peptides + KDO2	Melanoma helper peptides	KDO2-lipid A (TLR4 agonist)	Increased DC maturation and CD4^+^ T-cell activation	Salotto et al. (2022)
15	Nanoparticles with dual adjuvants (MPLA + CpG)	Tumor antigens (e.g., HER2, melanoma)	MPLA (TLR4) + CpG (TLR9)	High Th1 CD4^+^ and CD8^+^ activation; memory T-cell growth and tumor regression	Zupančič et al. (2018)

#### 5.3.4 Surface modification strategies for enhanced targeting and biocompatibility

A key component of NDDS optimization for targeted therapy and enhanced biocompatibility is surface modification. These changes are essential for minimizing toxicity and off-target effects while guaranteeing that nanocarriers can interact with target cells (like CD4^+^ T-cells) in a selective manner (Anwar et al., 2024). PEGylation is a common surface modification technique that entails affixing polyethylene glycol (PEG) chains to the surface of nanoparticles. By decreasing recognition by the mononuclear phagocyte system (MPS), this alteration lengthens the circulation time, delaying rapid clearance and boosting the nanoparticles’ therapeutic efficacy (Pershina et al., 2023). Another approach is ligand-functionalization, in which nanoparticles are coupled with targeting ligands like aptamers, peptides, or antibodies to enable them to bind specifically to particular receptors on CD4^+^ T-cells or tumor cells. For instance, anti-CD4^+^ antibody-coated nanoparticles can enhance CD4^+^ T-cell targeting, increasing the accuracy of immunotherapeutic approaches. The controlled release of drugs within the TME can also be facilitated by the use of pH-sensitive or enzyme-responsive coatings (Wang Y. et al., 2021). These changes guarantee that the nanocarriers release their payload precisely where the local conditions (like a lower pH or particular enzymes) cause it to do so. These surface alterations can increase NDDS’s biocompatibility, improving therapeutic treatments’ safety profile and targeting effectiveness. To fully realize these strategies’ potential in maximizing cancer immunotherapy through CD4^+^ T-cell modulation, future discussions should go into greater detail (Richardson et al., 2021).

## 6 Recent advances in nanoformulations for CD4^+^ T-cell immunotherapy

### 6.1 MHC II-coated nanoparticles for CD4^+^ T-cell expansion

Iron-dextran nanoparticles functionalized with MHC II molecules and co-stimulatory proteins (e.g., CD80, CD86) mimic antigen-presenting cells (APCs) to expand antigen-specific CD4^+^ T cells *ex vivo*. These artificial APCs (aAPCs) enhance CD4^+^ T-cell effector functions, including cytokine production (IFN-γ, IL-2) and cytotoxic activity against tumors. In murine models, these aAPCs improved CD8^+^ T-cell responses by promoting memory formation and tumor infiltration, leading to sustained antitumor immunity (Isser et al., 2022).

### 6.2 mRNA nanoformulations for dual CD4^+^/CD8^+^ T-cell priming

Lipid nanoparticles (LNPs) encapsulating mRNA vaccines (e.g., encoding tumor-associated antigens) activate dendritic cells (DCs) in lymphoid organs (Table 4). This approach primes the CD8^+^ cytotoxic T lymphocytes (CTLs) and CD4^+^ T-helper cells at the same time. Preclinical studies show that LNPs co-delivering antigen mRNA and immunostimulatory molecules (e.g., TLR agonists) enhance cross-presentation by DCs, fostering polyfunctional CD4^+^ T-cell responses critical for durable antitumor immunity (Conniot et al., 2014).

**TABLE 4:** Preclinical studies on nanoformulations for CD4^+^ T-Cell immunotherapy in cancer.

S. No.	Nanoformulation	Target/Mechanism	Key findings	References
1	MHC II-coated iron-dextran nanoparticles	Generate more CD4^+^ T-cells by imitating antigen-presenting cells (APCs)	Expanded antigen-specific murine CD4^+^ T-cellls with enhanced cytokine production (IFN-ƴ, IL-2) and antitumor activity in mouse models	Isser et al. (2022)
2	Immunoliposomes	Deliver TGF-β inhibitors to T cells	Enhanced granzyme expression in T-cells and reduced tumor growth in CNS models CD-90 targeted liposomes showed superior tumor suppression	Fang et al. (2022)
3	Polyethylenimine (PEI)- capped silica nanoparticles	Deliver TGF-β siRNA to suppress immunosuppression	Reduced Treg activity, increased Th1 polarization, and improved tumor control in lung cancer models	Babaei et al. (2017)
4	Lipid nanoparticles (LNPs) with mRNA vaccines	Co-deliver tumor antigens and immunostimulators 5(e.g., TLR agonists) to dendritic cells	Activated polyfunctional CD4^+^ T-cell responses, supporting durable CTL immunity	Conniot et al. (2014)
5	Gold nanoparticle- PEG hydrogels	Activate T cells via anti-CD3 antibodies and integrin-binding peptides	Enhanced *ex vivo* T-cell activation, proliferation, and memory formation. Improved adoptive T-cell therapy efficacy	Yesildag et al. (2018)
6	VEGF siRNA-loaded nanoparticles	Silence VEGF to remodel TME	Reduced angiogenesis and increased CD8^+^ T-cell infiltration in lung cancer models. Synergized with CD4^+^ T-cell responses	Butowska et al. (2023)
7	Chitosan-based nanoparticles	Delivery of checkpoint inhibitors to modify the immune response, such as anti-PD-1	Improved tumor control and immune checkpoint regulation result from increased CD4^+^ T-cell activation and proliferation	Timmins et al. (2020)
8	Polymeric nanocapsules (PLGA-based)	Delivery of siRNA to suppress Treg activity and modify immune cells	Enhanced anti-tumor immune responses, increased Th1 polarization, and decreased Treg activity	Sun et al. (2018)
9	DNA-loaded gold nanoparticles	Plasmid DNA containing tumor antigens is delivered	Increased tumor-specific immune response, effective antigen presentation, and stronger CD4^+^ T-cell activation	Vad et al. (2017)

### 6.3 Nanoparticle-mediated TGF-β suppression

Polyethylenimine (PEI)-capped silica nanoparticles loaded with TGF-β siRNA reduce immunosuppression in the TME. By silencing TGF-β, these nanoparticles decrease regulatory T-cell (Treg) activity and enhance CD4^+^ Th1 polarization, improving tumor control in lung cancer models (Khelil et al., 2022).

### 6.4 Immunoliposomes for T-cell activation

CD45- or CD90-targeted immunoliposomes encapsulating TGF-β inhibitors enhance adoptive T-cell therapy. Pre-incubation of T cells with these nanoparticles boosts granzyme expression and *in vivo* antitumor activity, particularly in central nervous system (CNS) tumor models (Conniot et al., 2014).

### 6.5 Clinical developments and clinical trails with NDDS

The table No. summarizes key clinical trials investigating nanoformulation -based drug delivery system concentrating on CD4^+^ T-cell reactions. Each trial highlights the use of different nanoparticle platforms designed to deliver specific tumor antigens or targets, aiming to enhance CD4^+^ T-cell activation and immune modulation (Lenders et al., 2020). The outcomes reported include improved helper T-cell responses, cytokine production, and overall immune support for anti-tumor activity (Table 5).

**TABLE 5:** Summary of clinical trials involving nanoparticle-based cancer targeting CD4^+^ T Cells.

S. No.	Trial ID	Nanoparticle type	Antigen/Target	Key CD4^+^ T-cell outcome	Phase/status	References
1	NCT03897881 N(Moderna mRNA-4157)	Lipid nanoparticles	Personalized neoantigens	Strong CD4^+^ Th1 responses; enhanced immune memory	Phase II (active)	Irvine and Dane (2020)
2	NCT04528641 (BioNTech BNT122)	Lipid nanoparticles	Patient-specific tumor neoantigens	Strong co-activation of CD4^+^ and CD8^+^ T cells in various malignancies	Phase II (active)	Pershina et al. (2023)
3	NCT03948763 (mRNA-5671)	Lipid nanoparticles	KRAS-mutated antigens	CD4^+^ T-helper support to CD8^+^ responses	Phase I (ongoing)	Wang et al. (2021a)
4	NCT04372706	Polymeric nanoparticles	OXO40 agonist + anti-CTLA-4	Amplified CD4^+^ activation and Th1 skewed response	Phase I (active)	Richardson et al. (2021)
5	NCT03436732	Synthetic polymeric nanoparticles	IL-2 tolerance modulation	Targeted Treg/CD4^+^ modulation; improved tumor tolerance response	Phase I (completed)	Yu et al. (2021)

#### 6.5.1 Adoptive transfer of nano-expanded CD4^+^ T cells

Under GMP settings, clinical experiments employing MHC II aAPCs show that tumor-specific CD4^+^ T cells can grow safely. In melanoma and lymphoma patients, these cells exhibit improved persistence and synergy with indigenous CD8^+^ T cells when co-administered with checkpoint inhibitors (e.g., anti-PD-1) (Yesildag et al., 2018).

#### 6.5.2 Neoantigen-targeted CD4^+^ T-cell vaccines

Personalized mRNA vaccines (e.g., Moderna’s mRNA-4157) encoding patient-specific neoantigens induce polyfunctional CD4^+^ T-cell responses. In early-phase trials, vaccinated patients exhibited durable clinical responses, with CD4^+^ T cells recognizing neoantigens absent in healthy tissues (Yu et al., 2022).

#### 6.5.3 Combination nano-immunotherapy

Trials combining nanoparticle-delivered OX40 agonists (CD4^+^ T-cell costimulators) with anti-CTLA-4 antibodies show improved tumor regression in advanced cancers. Nanoparticles localize OX40 agonists to tumor-draining lymph nodes, amplifying Th1 responses while minimizing systemic toxicity (Zhu and Li, 2023).

## 7 Challenges and limitations of nanoformulation-based CD4^+^ T-Cell therapy in cancer

Nanoformulation-based CD4^+^ T-cell therapies hold immense promise for cancer treatment by enhancing antitumor immune responses. However, several challenges limit their effectiveness and clinical translation. These challenges span biological barriers, stability issues, immunogenicity, and economic feasibility.

One of the critical hurdles in cancer immunotherapy is the ability to effectively deliver therapeutic agents to CD4^+^ T cells within the TME, which is highly immunosuppressive. Additionally, ensuring the stability and biocompatibility of nanoformulations while avoiding unintended immune responses remains a significant challenge. Finally, the high cost and scalability issues of these advanced therapies hinder their widespread adoption (Zhu and Li, 2023).

### 7.1 Biological barriers in CD4^+^ T-cell targeting

The TME presents formidable physical and immunological barriers that impede effective nanoparticle delivery to CD4^+^ T cells. Dense extracellular matrix (ECM), poor vascularization, and hypoxic conditions restrict nanoparticle penetration into tumors. For example, although MHC II-coated artificial antigen-presenting cells (aAPCs) have demonstrated efficacy in *ex vivo* expansion of antigen-specific CD4^+^ T cells, these obstacles restrict their *in vivo* delivery. Furthermore, when drug delivery systems enter the body, they can be unintentionally taken up by immune cells like macrophages. This process, known as nonspecific uptake, diverts the drug away from its intended target, such as tumor cells. As a result, the amount of drug reaching the tumor site is reduced. This not only lowers the treatment’s effectiveness but may also increase side effects in healthy tissues. Overcoming this challenge is crucial for improving the precision and success of targeted therapies. (Yu et al., 2022).

In CNS tumors, the blood-brain barrier (BBB) further complicates nanoparticle delivery. Immunoliposomes targeting CD45 or CD90 have shown promise in overcoming this issue by enhancing T-cell activation in CNS malignancies2. However, strategies like surface functionalization with tumor-specific ligands or size optimization (20–100 nm) are still required to improve lymph node trafficking and tumor infiltration.

### 7.2 Stability and biocompatibility of nanoformulations

Nanoformulations often face challenges related to stability during storage and circulation. Aggregation due to van der Waals forces or hydrophobic interactions alters nanoparticle size and distribution, leading to reduced efficacy. Protein corona formation—where serum proteins adsorb onto nanoparticle surfaces—can further alter their biological identity, leading to rapid clearance by the mononuclear phagocyte system (MPS) (Gavas et al., 2021).

Material toxicity is another concern. Inorganic nanoparticles like silica may induce oxidative stress, while polymeric carriers such as polyethylenimine (PEI) can cause cytotoxicity. To address these issues, surface modifications like PEGylation have been employed to improve stability and reduce immunogenicity. For example, PEG-functionalized carbon clusters have shown improved biocompatibility while targeting regulatory T cells (Tregs) in cancer models.

### 7.3 Potential side effects and immunogenicity of nanoparticles

Nanoparticles can elicit unintended immune responses that compromise their therapeutic potential. For instance, cytokine release syndrome (CRS) is a significant risk associated with overactivation of CD4^+^ T cells by MHC II aAPCs. Similarly, off-target effects may lead to autoimmunity or systemic inflammation when nanoparticles interact with non-cancerous tissues. Immunogenicity can also arise from the materials used in nanoparticle construction. Antibody-conjugated nanogels targeting CD4^+^ T cells may provoke anti-drug antibodies, reducing therapeutic efficacy over time5. Strategies such as using biodegradable materials or “stealth” coatings like polysaccharides are being explored to mitigate these risks (Zhu and Li, 2023; Lin et al., 2021).

### 7.4 Cost and scalability issues in clinical translation

One major obstacle to clinical translation is the expensive expense of producing nanoformulations under Good Manufacturing Practice (GMP) guidelines. Producing MHC II aAPCs or other complex nanoparticles involves rigorous quality control and specialized equipment, increasing costs by ∼30% compared to conventional therapies. Scalability is another major challenge. While modular platforms like plug-and-play aAPCs simplify production processes, reproducibility remains an issue for complex formulations such as cell-membrane-coated nanoparticles6. Microfluidic systems are being investigated for large-scale production but require further optimization for clinical use (Gavas et al., 2021).

### 7.5 Challenges and limitations: negative findings, off-target effects, and clinical trial failures

Although NDDS has great potential for cancer immunotherapy, expectations should be moderated due to a number of obstacles and contradictory findings in preclinical and clinical settings (Becht et al., 2024). The possibility of off-target effects, in which nanoparticles build up in tissues they are not intended to reach and may become toxic, is an important concern. For example, some nanoparticles have demonstrated a propensity to unintentionally activate immune cells that are not their intended target, like macrophages (Sousa-Ju et al., 2022). This can lead to inflammatory reactions or unintentional suppression of the intended immune activation. Furthermore, because nanoparticles are highly biocompatible and simple to surface functionalize, they occasionally cause unexpected interactions in the TME that alter immune responses and reduce the efficacy of treatment (Wang et al., 2023). Additionally, although a number of preclinical investigations have demonstrated encouraging outcomes in terms of modifying CD4^+^ T-cell responses with NDDS, it has proven more difficult to convert these results into fruitful clinical outcomes (Turtle, 2017). Results from clinical trials using checkpoint inhibitors and mRNA nanovaccines administered via nanoparticles have been inconsistent; some have shown insufficient immune response activation or limited tumor regression (Hongxia et al., 2019). The inability to accurately target all CD4^+^ T-cell subsets, the complexity of the TME, and the possibility of quick immune system clearance of nanoparticles continue to be major obstacles. These elements emphasize the necessity of more thorough investigation into the long-term safety and effectiveness of nanoparticle formulations in human trials, as well as more rigorous optimization of those formulations (Xiang et al., 2023).

### 7.6 NDDS and their manufacturing cost-benefit analysis with manufacturing challenges

A critical cost-benefit analysis of NDDS is necessary to assess the clinical translation of these systems in addition to their promising therapeutic benefits. Significant difficulties arise from the high cost of producing complex nanoformulations and the difficulty of manufacturing them under GMP guidelines (Su et al., 2022). Additionally, the commercial viability of customized nanocarriers or those that require complex surface modifications may be limited by the scale at which they can be produced. Making NDDS-based treatments more widely available in clinical settings may depend on resolving these problems through cost-cutting measures or more effective manufacturing techniques (Anwar et al., 2024).

### 7.7 Toxicity considerations in NDDS-based CD4^+^ T-cell immunotherapy

Since these systems interact with the immune system and other body tissues, a critical evaluation of toxicity is in fact necessary when evaluating NDDS for therapeutic purposes. NDDS have the benefit of targeted delivery, but they may also carry some toxicity risks, especially when it comes to accumulation in non-target organs, immune system activation, and off-target effects (Milling et al., 2017). Some nanocarriers, like liposomes and polymeric nanoparticles, can cause cytotoxicity or trigger inflammatory reactions, especially if they build up in vital organs like the kidneys or liver. Moreover, PEGylation and other surface alterations that improve targeting may also trigger immunological reactions that jeopardize the effectiveness of treatment (Li et al., 2022; Estapé et al., 2022). Furthermore, there are still questions about the long-term safety of NDDS due to the possibility of toxic byproducts being released from the degradation of nanomaterials and chronic exposure (Fadeel et al., 2017). Additionally, the immune system may become resistant to repeated nanoparticle dosages, decreasing their efficacy and possibly leading to allergic or hypersensitive reactions. To guarantee the safety of NDDS in CD4^+^ T-cell-based immunotherapy, future research should concentrate on enhancing the biocompatibility of nanocarriers, refining dosage schedules, and closely observing toxicological profiles in preclinical and clinical trials (El-Sayed et al., 2015; Mohammapdour and Ghandehari, 2022).

## 8 Future directions and perspectives in nanoformulation-based cancer immunotherapy

Nanoformulations are revolutionizing cancer immunotherapy by enabling precision targeting, enhancing delivery mechanisms, and integrating computational tools for therapy optimization. Emerging strategies focus on personalized approaches, smart nanocarriers, and artificial intelligence (AI)-driven advancements (Gao et al., 2021).

### 8.1 Personalized nanoformulation strategies for cancer immunotherapy

Personalized cancer immunotherapy leverages nanotechnology to tailor treatments based on individual tumor profiles. Lipid nanoparticles (LNPs) encapsulating mRNA encoding patient-specific neoantigens have shown promise in activating CD4^+^ helper T cells and CD8^+^ cytotoxic T lymphocytes (CTLs). These vaccines target unique tumor antigens identified through genomic sequencing, inducing robust immune responses. Additionally, metal-organic frameworks (MOFs) combine tumor-killing agents with immune stimulators, addressing tumor heterogeneity while boosting immune recognition.

Dendritic cell-based vaccines also exemplify personalized strategies, where patient-derived dendritic cells are loaded *ex vivo* with tumor antigens to stimulate adaptive immunity. Provenge^®^, A prostate cancer vaccine approved by the FDA, demonstrates the feasibility of such approaches despite challenges like labor-intensive manufacturing and high costs (Thirumalai et al., 2024).

### 8.2 Smart nanocarriers with controlled release mechanism

Smart nanocarriers improve drug bioavailability and minimize systemic toxicity by releasing therapeutic drugs in reaction to particular stimuli, including pH shifts or hypoxia, within the TME. Polymeric nanoparticles with pH-sensitive coatings release immunomodulators only in acidic environments typical of tumors. Self-assembling micelles and scaffolds have been developed for localized delivery of immune checkpoint inhibitors (e.g., anti-PD-1/PD-L1 antibodies), enhancing efficacy while reducing off-target effects.

Dual-release mechanisms in nanocarriers enable combination therapies by co-delivering immune checkpoint inhibitors and tumor antigens, synergistically reversing immune evasion and activating T cells (Wang J. et al., 2021).

### 8.3 Role of artificial intelligence in nanomedicine development

AI and machine learning (ML) optimize nanoformulations by predicting nanoparticle interactions with immune cells and the TME. These technologies accelerate development by simulating biodistribution, stability profiles, and therapeutic efficacy under varying physiological conditions. AI-driven platforms identify optimal combinations of nanomaterials, payloads, and targeting ligands for specific cancer types while facilitating patient stratification based on biomarkers indicating responsiveness to nano-immunotherapy.

AI also enhances scalability by optimizing manufacturing processes for consistent production under GMP conditions, reducing costs while ensuring quality (Skepu et al., 2023).

## 9 Conclusion

This review highlights the critical role of CD4^+^ T-cells in cancer immunology, demonstrating their dual function as both tumor-suppressing and tumor-promoting agents. The dynamic interplay of CD4^+^ T-cell subsets, including Th1, Th2, Th17, Tregs, and Tfh, significantly influences the TME and patient outcomes. Targeting CD4^+^ T-cells with NDDS has become a viable tactic to improve treatment efficacy as immunotherapy advances. Nanoparticle-based formulations, including liposomes, polymeric nanoparticles, dendrimers, and metallic nanoparticles, have shown remarkable potential in modulating CD4^+^ T-cell responses, providing targeted delivery of cytokines, antigens, and immune checkpoint inhibitors. The advantages of NDDS include improved bioavailability, controlled release, reduced systemic toxicity, and enhanced targeting in the TME of CD4^+^ T-cells. Specific applications, such as MHC II-coated nanoparticles for CD4^+^ T-cell expansion, mRNA vaccines for dual CD4^+^/CD8^+^ activation, and TGF-β suppression with silica nanoparticles, demonstrate the versatility of NDDS in enhancing antitumor immunity. However, challenges persist, including biological barriers, stability, immunogenicity, and scalability issues, which limit the clinical translation of NDDS-based CD4^+^ T-cell therapies.

Future research should prioritize overcoming these challenges by optimizing nanoparticle design, improving targeting specificity, and integrating personalized medicine approaches. Additionally, leveraging artificial intelligence (AI) for nanoparticle optimization and combining NDDS with conventional therapies may further enhance therapeutic outcomes. By bridging the gap between nanotechnology and immunology, NDDS-based CD4^+^ T-cell
